# The Gut Microbiota‐Xanthurenic Acid‐Aromatic Hydrocarbon Receptor Axis Mediates the Anticolitic Effects of Trilobatin

**DOI:** 10.1002/advs.202412234

**Published:** 2025-01-21

**Authors:** Xiaoyu Wu, Jiajia Wei, Wang Ran, Dongjing Liu, Yang Yi, Miaoxian Gong, Xin Liu, Qihai Gong, Haibo Li, Jianmei Gao

**Affiliations:** ^1^ Key Laboratory of Basic Pharmacology of Ministry of Education and Joint International Research Laboratory of Ethnomedicine of Ministry of Education Department of Pharmacology Key Laboratory of Basic Pharmacology of Guizhou Province and School of Pharmacy Zunyi Medical University Zunyi 563000 China; ^2^ Faculty of Functional Food and Wine Shenyang Pharmaceutical University Shenyang 110016 China; ^3^ School of Traditional Chinese Medicine Liaoning University of Traditional Chinese Medicine Shenyang 110016 China

**Keywords:** aromatic hydrocarbon receptor, gut microbiota, trilobatin, ulcerative colitis, xanthurenic acid

## Abstract

Current treatments for ulcerative colitis (UC) remain limited, highlighting the need for novel therapeutic strategies. Trilobatin (TLB), a naturally derived food additive, exhibits potential anti‐inflammatory properties. In this study, a dextran sulfate sodium (DSS)‐induced animal model is used to investigate the effects of TLB on UC. It is found TLB significantly alleviates DSS‐induced UC in mice, as evidenced by a reduction in the disease activity index, an increase in colon length, improvement in histopathological lesions. Furthermore, TLB treatment results in a decrease in proinflammatory cytokines and an increase in anti‐inflammatory cytokines. TLB mitigates UC by modulating the intestinal microbiota, particularly *Akkermansia*, which enhances tryptophan metabolism and upregulates the production of xanthurenic acid (XANA). To confirm the role of TLB‐induced microbiota changes, experiments are performed with pseudogerm‐free mice and fecal transplantation. It is also identified XANA as a key metabolite that mediates TLB's protective effects. Both TLB and XANA markedly activate the aromatic hydrocarbon receptor (AhR). Administration of an AhR antagonist abrogates their protective effects, thereby confirming the involvement of AhR in the underlying mechanism. In conclusion, the study reveals a novel mechanism through which TLB alleviates UC by correcting microbiota imbalances, regulating tryptophan metabolism, enhancing XANA production, and activating AhR.

## Graphical abstract

Trilobatin (TLB) acts as an AhR ligand, directly activating AhR, suppressing intestinal inflammation, and facilitating intestinal homeostasis. Moreover, the activation of AhR by TLB enhances AhR expression by regulating the gut microbiota and its metabolite xanthurenic acid, forming a positive feedback loop. These discoveries highlight the dual roles of TLB, both in directly binding to AhR and indirectly modulating its expression and activity through microbiota interactions.

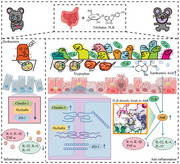

## 1 Introduction

Ulcerative colitis (UC) is a chronic, relapsing intestinal disease classified under inflammatory bowel diseases.^[^ ^1^ ^]^ UC primarily affects the mucosa of the colon and rectum, characterized by chronic nonspecific inflammation, dysbiosis of the gut microbiota, epithelial barrier dysfunction, immune dysregulation, and an increased risk of colorectal cancer.^[^ ^2^ ^]^ The pathogenesis of UC is complex and involves the interplay of various factors, including genetics, environment, immune responses, and gut microbiota.^[^ ^3^ ^]^ Currently, our understanding of the prevention and treatment of UC is limited. Clinical treatments mainly focus on symptom relief with medications, such as sulfasalazine, but secondary failures or severe adverse effects often occur.^[^ ^4^ ^]^ Therefore, there is an urgent need for more effective and safer therapeutic interventions.

The colon harbors a dynamic and complex microbial community known as the gut microbiota, which plays a crucial role in human health and disease.^[^ ^5^ ^]^ Despite the unclear pathogenesis of UC, mounting evidence suggests that the microbiome plays a pivotal role in regulating gut homeostasis.^[^ ^6^ ^]^ Currently, intervening with specific gut strains or enhancing the gut microbial ecology to maintain the integrity of the intestinal barrier and normal permeability is a promising approach for UC treatment.^[^ ^7^ ^]^ Studies have shown that dysbiosis in UC patients is characterized by a significant reduction in tryptophan‐metabolizing bacteria, such as *Akkermansia* (AKK), leading to inflammation and mucosal lesions in colitis.^[^ ^8^ ^]^ More crucially, recent research indicates that the gut microbiota can mitigate intestinal inflammation in colitis‐afflicted mice by generating beneficial indole metabolites through the regulation of tryptophan (Trp) metabolism.^[^ ^9^ ^]^ Trp metabolism primarily involves three metabolic pathways kynurenine (Kyn), indole, and 5‐hydroxytryptamine pathways.^[^ ^10^ ^]^ The major Trp metabolism pathway is the Kyn pathway; through this pathway, >95% of Trp degrades into multiple bioactive compounds. The Kyn pathway leads to the production of several end‐metabolites, including xanthurenic acid (XANA), kynurenic acid, and quinolinic acid.^[^ ^11^ ^]^ Notably, some metabolites of the Kyn pathway exert their effects on immune cells primarily through the activation of the aromatic hydrocarbon receptor (AhR), which plays a crucial role in T cell differentiation, the production of IL‐22, and the maintenance of type 3 innate lymphoid cells or antioxidant responses in the gut.^[^ ^12^ ^]^ Therefore, the gut microbiota and its microbial metabolites, especially indole derivatives, constitute a promising pool for the development of novel therapeutic approaches for UC, and targeting intestinal homeostasis might be feasible for the improvement of UC.

Increasing evidence underscores the pharmacological benefits of natural medicinal resources. The use of traditional Chinese medicine and herbal medicine, known for their health benefits and minimal side effects, has become a promising approach for disease management.^[^ ^13^ ^]^ Trilobatin (TLB) is a natural sweetener derived from the plant‐based sweet tea, known for its numerous pharmacological properties.^[^ ^14^ ^]^ Our studies suggest that TLB improves alcoholic liver disease and inhibits age‐induced cognitive impairment by regulating the gut microbiota.^[^ ^15^ ^]^ Although TLB could protect against UC through modulating gut microbiota,^[^ ^16^ ^]^ the detailed mechanism or potential therapeutic targets of TLB on UC still unclear.

This study aims to evaluate the anti‐UC activity of TLB and to elucidate the pivotal role of gut microbiota in mediating its effects through multiomics analysis and fecal microbiota transplantation (FMT). We demonstrated that TLB mitigated intestinal inflammation by directly targeting the AhR. Furthermore, we identified a key active metabolite, XANA, as a mediator of TLB's beneficial effects on ulcerative colitis, primarily via activation of the AhR signaling pathway. This research not only enhances our understanding of TLB's therapeutic potential but also establishes a scientific foundation for future investigations into TLB and gut microbiota‐based therapies for UC.

## 2 Result

### 2.1 TLB Alleviates DSS‐Induced UC by Reducing Intestinal Inflammation in Mice

**Figure 1:**
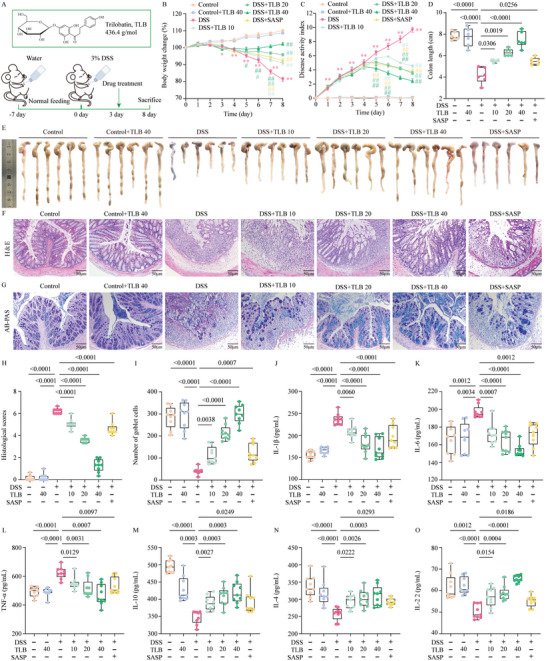
TLB treatment alleviates DSS‐induced UC in C57BL/6 mice. A) Schematic of the animal experiment 1. B) Body weight change (%) of mice (*n* = 9). C) DAI score (*n* = 9). D) Colon length (*n* = 6). E) Representative images of the colonic tissue. F) Representative images of colonic sections stained with H&E (scale bars = 50 µm). G) Representative images of colonic sections stained with AB‐PAS (scale bars = 50 µm). H) Histological scores (*n* = 9). I) Number of goblet cells (*n* = 9). J) IL‐1β (*n* = 9). K) IL‐6 (*n* = 9). L) TNF‐α (*n* = 9). M) IL‐10 (*n* = 9). N) IL‐4 (*n* = 9). O) IL‐22 (*n* = 9). ^*^ *P* < 0.05, ^**^ *P* < 0.01 versus control group; ^#^ *P* < 0.05, ^##^ *P* < 0.01 versus DSS group.

We first investigated the anti‐UC effects of TLB in DSS‐induced UC mice (**Figure** **1**A). Over the course of the experiment, DSS mice exhibited progressive weight loss and higher DAI scores (Figure 1B,C), compared to control group. TLB and SASP treatment significantly mitigated weight loss, improved diarrhea and rectal bleeding, and reduced DAI scores. Moreover, DSS induced severe pathological changes in the colon, characterized by a significant reduction in colon length (*P* < 0.001) (Figure 1D,E), damage to crypt and colon tissue structure (Figure 1F,H), loss of goblet cells (Figure 1G,I), and marked inflammation (including increased IL‐1β, IL‐6, TNF‐α, and decreased IL‐4, IL‐10, and IL‐22) (Figure 1J–O). TLB at various doses (10, 20, and 40 mg kg^−1^) alleviated these pathological symptoms, indicated by weight gain and improved colon length and structure. Additionally, TLB restored the number of goblet cells and reversed cytokine level changes in a dose‐dependent manner. Interestingly, the lower doses of TLB provided significantly better protection against colonic damage compared to the positive drug (SASP) demonstrating its in vivo anti‐UC activity.

### 2.2 TLB Improves Intestinal Barrier Function by Activating AhR

**Figure 2:**
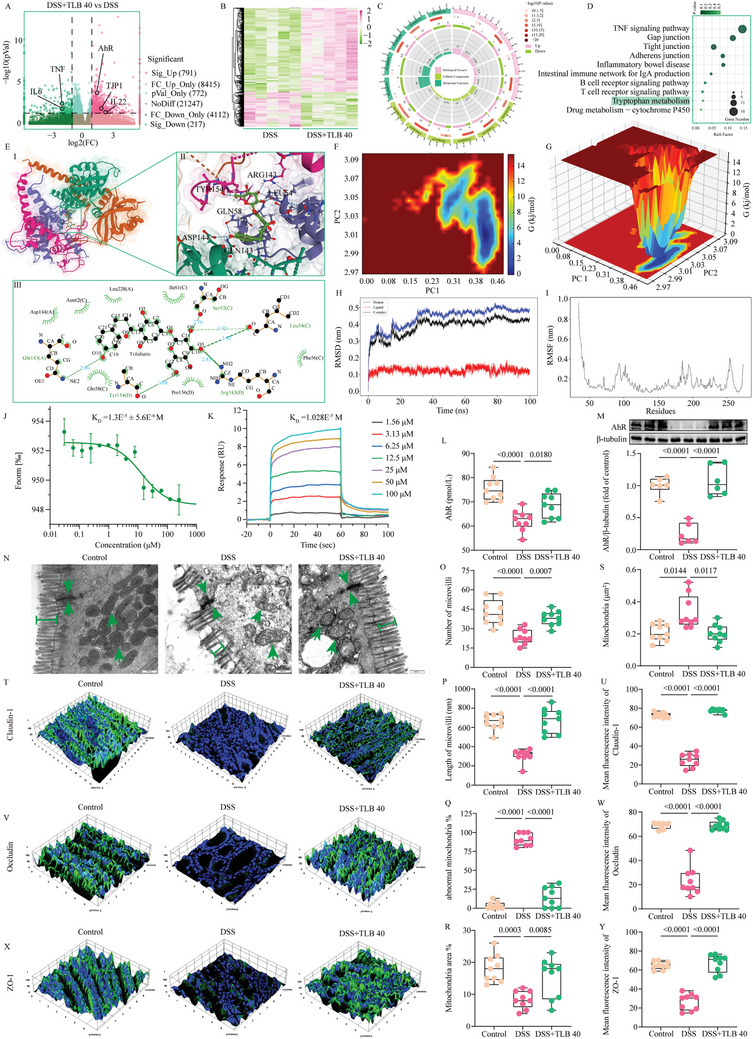
TLB restores intestinal barrier homeostasis by activating AhR. A) Volcano plot of DSS+TLB 40 mg kg^−1^ versus DSS group (*n* = 5). B) Heatmap of DEGs. C) GO enrichment analysis of DEGs. D) KEGG pathway analysis of DEGs. E) TLB directly bound to AhR, I) The entire view of the binding sites between TLB onto AhR; II) A close‐up view of the molecular binding pocket; III) 2D interaction map for TLB onto AhR protein binding site. F) Gibbs energy landscape (2D). G) Gibbs energy landscape (3D). H) RMSD. I) RMSF. J) MST analysis of TLB binding to AhR. K) SPR analysis of TLB binding to AhR. L) ELISA analysis of AhR (*n* = 9). M) WB analysis of AhR (*n* = 6). N) Representative TEM images of colonic tissue (scale bars = 500 nm). O) Number of microvilli (*n* = 9). P) Length of microvilli (nm, *n* = 9). Q) % of abnormal mitochondria (*n* = 9). R) % of Mitochondria area (*n* = 9). S) Mitochondria (µm^2^, *n* = 9). T) IF imaging of tight junction structures using an antibody against Claudin‐1. U) Mean fluorescence intensity of Claudin‐1 (*n* = 9). V) IF imaging of tight junction structures using an antibody against Occludin. W) Mean fluorescence intensity of Occludin (*n* = 9). X) IF imaging of tight junction structures using an antibody against ZO‐1. Y) Mean fluorescence intensity of ZO‐1 (*n* = 9).

To explore the potential mechanisms of TLB's protective effect against DSS‐induced UC, we conducted transcriptomic analysis of colonic RNA levels in the DSS and DSS+TLB 40 mg kg^−1^ groups (*n* = 5). PCA results showed significant differences between DSS and DSS+TLB 40 mg kg^−1^ groups (Figure S1A, Supporting Information). Differentially expressed genes (DEGs) were presented using volcano plots and heatmaps. Compared to the DSS group, 791 genes were significantly upregulated (e.g., AhR, TJP1, and IL22) and 217 genes were significantly downregulated (e.g., IL‐6 and TNF) following TLB intervention (**Figure** **2**A,B). GO analysis of DEGs revealed enrichment in pathways, such as Negative regulation of inflammatory response (GO:00 50728), Microvillus (GO:0 005902), Cell junction (GO:00 30054), and Trp catabolic process to kynurenine (GO:00 19441) (Figure 2C). KEGG analysis indicated significant enrichment in the TNF signaling pathway, Tight junction, and Trp metabolism, which was consistent with the above results (Figure 2D). Based on these findings, we used molecular docking and MD simulation to predict the interaction between TLB and AhR. The results indicated that TLB binds directly to AhR through residues such as Gln143, Tyr154, Arg143, Leu54, and Ser52, with a binding energy of −7.899 kcal mol^−1^ (Figure 2E). Further MD simulation showed that the interaction between TLB and AhR was stable, and the binding of TLB made AhR molecules more compact, reduced the surface area, and formed many hydrogen bonds with small molecules (Figure 2F–I). MST and SPR assay was used to verify the interaction between TLB and recombinant human AhR. MST assay was performed to determine the direct binding between TLB and AhR. As expected, TLB readily bound to AhR proteins with equilibrium dissociation constant (*K*_D_) estimated at 1.3E^−5^ ± 5.6E^−6^ M (Figure 2J). Furthermore, the *K*_D_ values for TLB bound to AhR based on SPR analysis was determined to be 1.028E^−5^ M (Figure 2K). These results indicate that TLB displays a stronger binding affinity and directly toward to AhR. Additionally, we measured AhR levels in colonic tissues using ELISA and WB. Compared to the control group, AhR levels were significantly downregulated in the DSS group, while TLB 40 mg kg^−1^ intervention markedly upregulated AhR levels (Figure 2L,M). TEM revealed significant ultrastructural damage in the DSS group compared to the control group, including increased tight junction gaps, reduced and shortened microvilli, and mitochondrial damage (Figure 2N–S). TLB 40 mg kg^−1^ treatment significantly reversed these damages. IF staining further showed that tight junction proteins (Claudin‐1, Occludin, ZO‐1) were significantly downregulated in the DSS group compared to the control group (*P* < 0.001). However, TLB 40 mg kg^−1^ intervention significantly restored the levels of Claudin‐1, Occludin, and ZO‐1 (*P* < 0.001) (Figure 2T–Y; and Figure S1B, Supporting Information).

### 2.3 TLB Ameliorates Gut Microbiota Dysbiosis and Increases *AKK* Abundance

**Figure 3:**
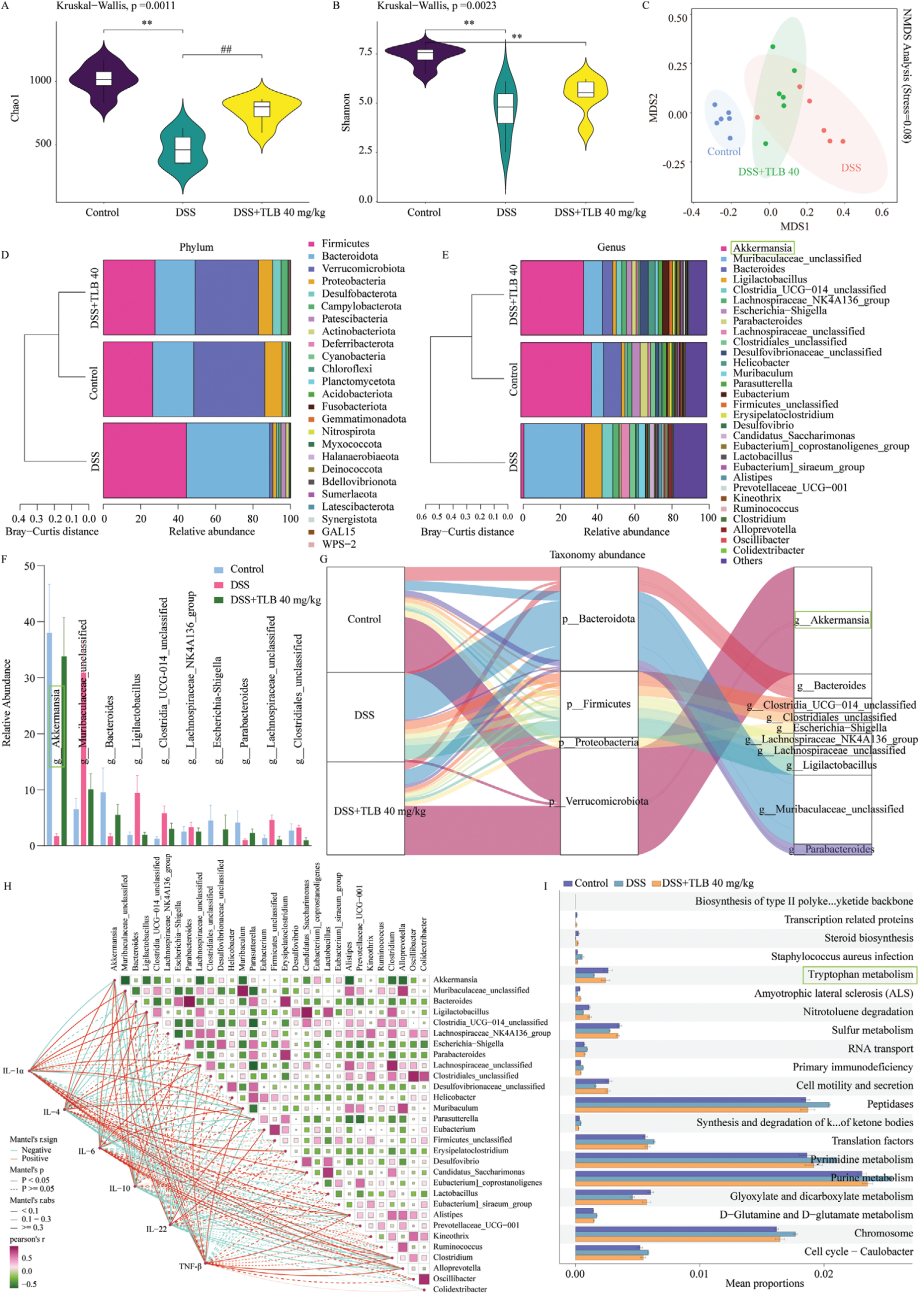
TLB ameliorates gut microbiota dysbiosis in mice with DSS‐induced UC. Microbial α diversity was assessed by the Chao1 index A) and Shannon index B). C) Microbial β diversity was assessed by NMDS. Bacterial taxonomic profiling at the phylum D) and geuns E) levels. F) Bacterial taxonomic profiling at top 10 most abundant genera. G) Sankey diagram. H) Correlation analysis. I) Functions of microbial gene. *n* = 6, ^**^ *P* < 0.01 versus control group; ^##^ *P* < 0.01 versus DSS group.

We conducted 16S rRNA gene sequencing to evaluate the impact of TLB treatment on the gut microbiota. The alpha diversity of the gut microbiota in DSS‐induced colitis mice was significantly lower than that in normal mice (*P* < 0.01). TLB 40 mg kg^−1^ supplementation significantly increased alpha diversity, as indicated by Chao1 and Shannon indices (**Figure** **3**A,B). Nonmetric multidimensional scaling (NMDS) analysis showed that TLB treatment shifted the gut microbiota composition toward that of the control group (Figure 3C). Additionally, TEM revealed that TLB treatment increased the number of gut microbiota (Figure S2A, Supporting Information). At the phylum level, compared to the control group, the abundance of *Firmicutes* and *Bacteroidetes* decreased in the DSS group, while the abundance of *Verrucomicrobiota* and *Proteobacteria* increased. TLB reversed these changes, bringing the microbial abundance closer to that of the control group (Figure 3D). At the genus level, the abundance of *AKK*, *Bacteroides*, and *Parabacteroides* decreased in the DSS‐treated mice, while the abundance of *Muribaculaceae*, *Clostridia*, *Lachnospiraceae*, and *Escherichia‐Shigella* increased, all of which were reversed by TLB treatment (Figure 3E,F). Linear discriminant analysis (LDA) effect size (LEfSe) analysis showed that *AKK* was the dominant flora in the control group (Figure S2B, Supporting Information). A sankey diagram showed changes in the relative abundance of species between the phylum and genus levels, indicating that TLB significantly modulates the gut microbiota composition in DSS‐induced colitis mice (Figure 3G). Correlation analysis between genus‐level microbiota and inflammatory factors revealed that *AKK*, *Muribaculum*, and *Alloprevotella* were negatively correlated with proinflammatory cytokines and positively correlated with anti‐inflammatory cytokines (*P* < 0.05, Figure 3H). Using PICRUSt 2, we mined the 16S RNA sequencing data to predict potential functions of microbial genes, aiming to preliminarily explore the link between dysbiosis and disease. The results suggested that TLB‐modulated microbiota exert their effects primarily by altering metabolic pathways related to colitis, particularly Trp metabolism, sulfur metabolism, and glyoxylate and dicarboxylate metabolism (Figure 3I).

### 2.4 TLB Alters Gut Microbiota‐Derived Tryptophan Metabolism in DSS‐Induced UC Mice

**Figure 4:**
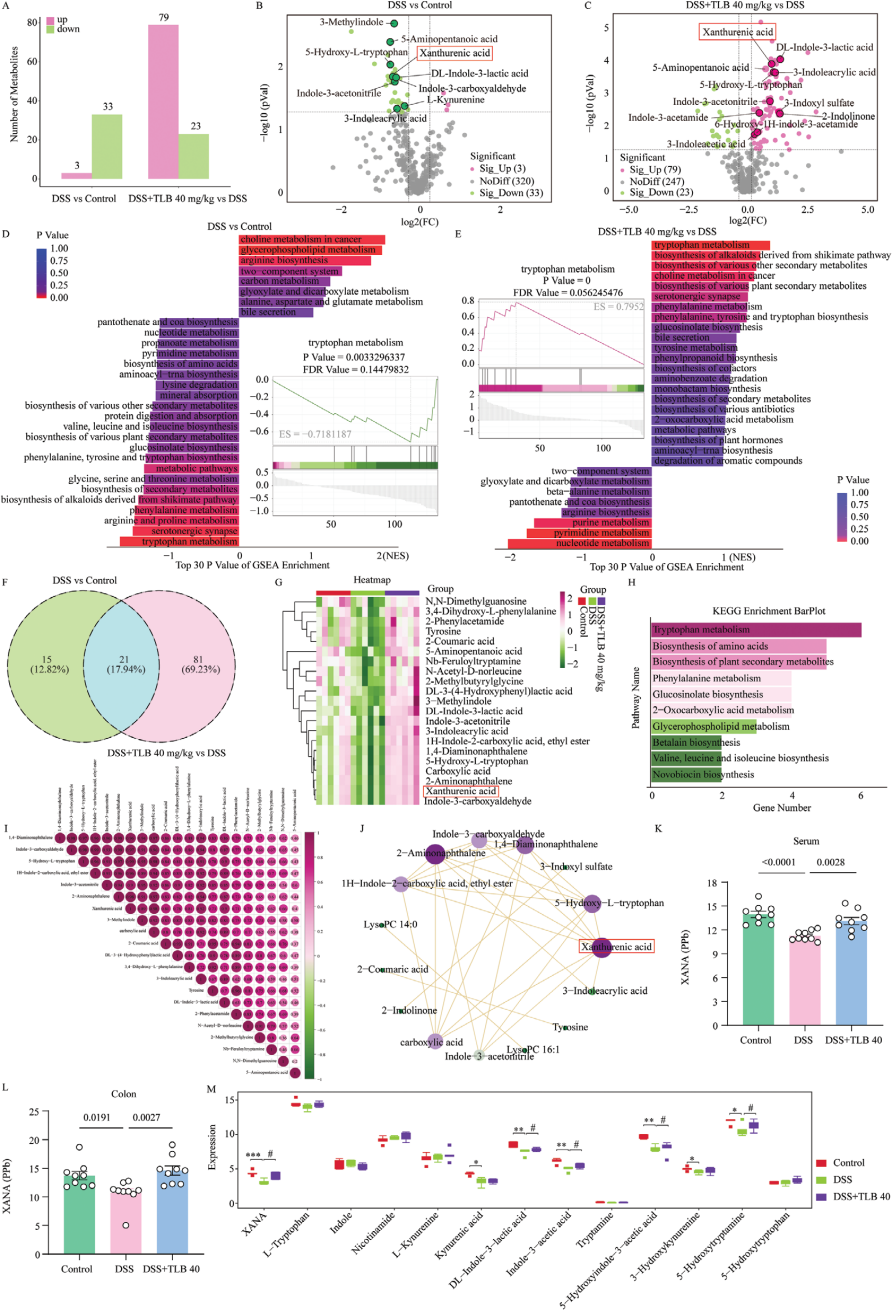
TLB alters gut microbiota‐derived tryptophan metabolism in DSS‐induced UC mice. A) Number of metabolites. B) Volcano plot of DSS versus control (*n* = 6). C) Volcano plot of DSS+TLB 40 mg kg^−1^ versus DSS (*n* = 6). D) GSEA enrichment of DSS versus control. E) GSEA enrichment of DSS+TLB 40 mg kg^−1^ versus DSS. F) Venn of metabolites. G) Heatmap of metabolites. H) KEGG enrichment of metabolites. I) Correlation heatmap of metabolites. J) Correlation network of metabolites. K) ELISA analysis of XANA in serum (*n* = 9). L) ELISA analysis of XANA in colon (*n* = 9). M) Quantification of Trp and its metabolites in mice serum (*n* = 5). ^**^ *P* < 0.01, ^***^ *P* < 0.001 versus control group; ^#^ *P* < 0.05 versus DSS group.

Given the regulatory effects of TLB on the gut microbiome, we performed metabolomics analysis to identify functional microbial metabolites. Compared to untreated mice, DSS‐induced colitis significantly altered metabolite levels, while TLB treatment notably restored the distribution of these metabolites (**Figure** **4**A). Differential analysis was conducted with thresholds set at ratio > 1.5 or ratio < 1/1.5, *Q* value < 0.05, and VIP > 1 (*n* = 6). In the comparison between the DSS and control groups, 3 differential metabolites increased and 33 decreased (Figure 4B; and Figure S3A, Supporting Information). Between the DSS + TLB 40 mg kg^−1^ and DSS groups, 79 metabolites increased, and 23 decreased (Figure 4C; and Figure S3B, Supporting Information). Subsequent GSEA enrichment analysis of the differential metabolites revealed that those in DSS and control exacerbated UC through downregulated pathways like Trp metabolism, Serotonergic synapse, and Arginine and proline metabolism, whereas TLB treatment in DSS + TLB 40 mg kg^−1^ and DSS reversed the regulation of these pathways (Figure 4D,E). Additionally, 21 shared differential metabolites were identified between the two comparisons (Figure 4F). A heatmap illustrated the abundance of these 21 metabolites across the groups, showing that Trp metabolites and indole derivatives were significantly downregulated in the DSS group compared to the control group. TLB treatment restored the abundance of these metabolites, particularly XANA (Figure 4G). KEGG enrichment analysis of the differential metabolites highlighted Trp metabolism as the main pathway, consistent with microbiome results (Figure 4H). Correlation analysis indicated that XANA is a key differential metabolite, potentially playing an important role in TLB's amelioration of UC (Figure 4I,J). In addition, the content of XANA in serum and colon was determined by ELISA, which was in line with the aforementioned findings. The results showed that TLB significantly restored the content of XANA in serum and colon (Figure 4K,L). Meanwhile, we used targeted metabolomics to detect alterations in Trp metabolites associated with gut microbiota in mouse serum, specifically focusing on changes in XANA. The results showed that the serum XANA in the model group was decreased (*P* < 0.001) compared to the control group. Furthermore, the concentrations of indole derivatives, including Kynurenic Acid, DL‐Indole‐3‐Lactic Acid, Indole‐3‐Acetic Acid, 5‐Hydroxyindole‐3‐Acetic Acid, 3‐Hydroxykynurenine, and 5‐Hydroxytryptamine, exhibited a reduction (*P* < 0.05, Figure 4M). However, compared to the DSS group, TLB treatment reversed the levels of these Trp metabolites. These results imply that TLB may exert therapeutic effects by regulating gut microbiota‐derived tryptophan metabolism, especially XANA.

### 2.5 TLB‐Regulated Gut Microbiota Restores Intestinal Barrier Function by Upregulating XANA

**Figure 5:**
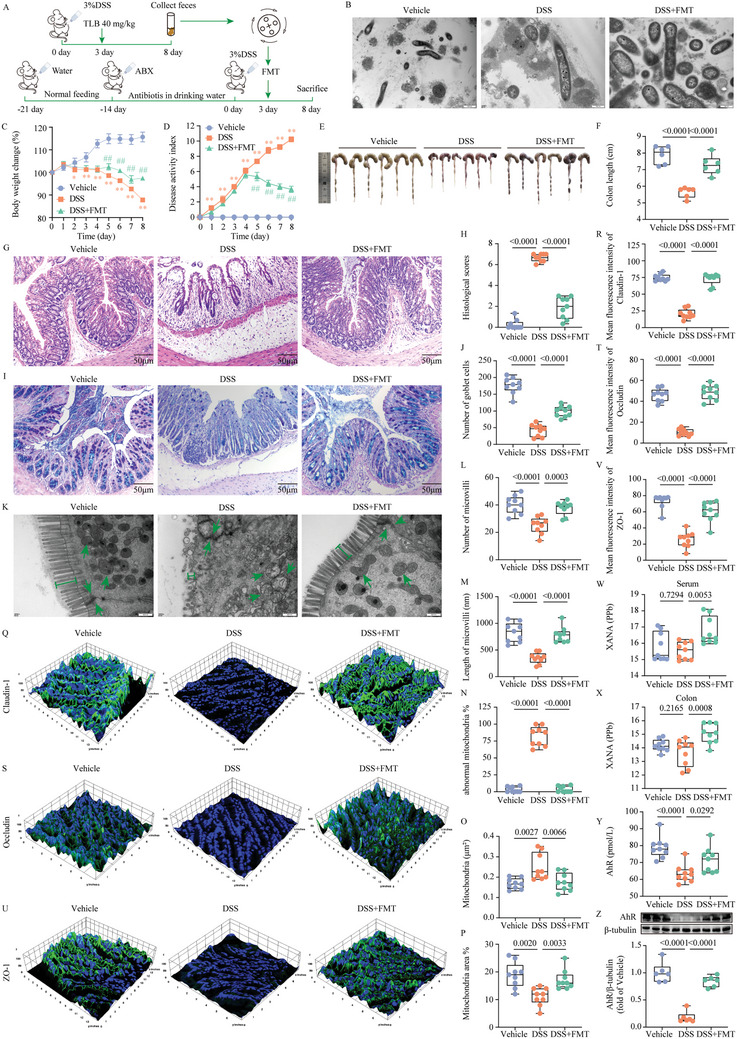
TLB‐regulated gut microbiota restores intestinal barrier function by upregulating XANA. A) Schematic of the animal experiment 2. B) Representative TEM images of gut microbiota (scale bars = 500 nm). C) Body weight change (%) of mice (*n* = 9). D) DAI score (*n* = 9). E) Representative images of colonic tissue. F) Colon length (*n* = 6). G) Representative images of colonic sections stained with H&E (scale bars = 50 µm). H) Histological scores (*n* = 9). I) Representative images of colonic sections stained with AB‐PAS (scale bars = 50 µm). J) Number of goblet cells (*n* = 9). K) Representative TEM images of colonic tissue (scale bars = 500 nm). L) Number of microvilli (*n* = 9). M) Length of microvilli (nm, *n* = 9). N) % of abnormal mitochondria (*n* = 9). O) Mitochondria (µm^2^, *n* = 9). P) % of Mitochondria area (*n* = 9). Q) IF imaging of tight junction structures using an antibody against Claudin‐1. R) Mean fluorescence intensity of Claudin‐1 (*n* = 9). S) IF imaging of tight junction structures using an antibody against Occludin. T) Mean fluorescence intensity of Occludin (*n* = 9). U) IF imaging of tight junction structures using an antibody against ZO‐1. V) Mean fluorescence intensity of ZO‐1 (*n* = 9). W) ELISA analysis of XANA in serum (*n* = 9). X) ELISA analysis of XANA in colon (*n* = 9). Y) ELISA analysis of AhR (*n* = 9). Z) WB analysis of AhR (*n* = 6). ^*^ *P* < 0.05, ^**^ *P* < 0.01 versus vehicle group; ^##^ *P* < 0.01 versus DSS group.

To further evaluate whether the gut microbiota regulated by TLB is sufficient to produce anti‐UC effects, we constructed a germ‐free colitis mouse model by administering ABX to mice prior to DSS treatment. We then transplanted feces from TLB‐treated UC mice into the DSS‐induced pseudogerm‐free mice model (**Figure** **5**A). TEM observation of gut microbiota revealed a significant reduction in microbial numbers in both the Vehicle and DSS groups, indicating successful microbiota depletion. In contrast, the DSS+FMT group showed a significant increase in microbial numbers (Figure 5B). In addition, FMT treatment significantly mitigated weight loss, improved diarrhea and rectal bleeding, and reduced DAI scores (Figure 5C,D). Additionally, compared to DSS mice, FMT mice exhibited normal trends in colon length and histology (Figure 5E–J). Furthermore, TEM revealed significant ultrastructural damage in the DSS group compared to the Vehicle group, including increased tight junction gaps, reduced and shortened microvilli, and mitochondrial damage (Figure 5K–P). FMT treatment significantly reversed these damages. Notably, the weakened intestinal barrier in UC mice was significantly restored after transplanting TLB‐regulated gut microbiota, as reflected in the upregulation of Claudin‐1, Occludin, and ZO‐1 expression (*P* < 0.001) (Figure 5Q–V; and Figure S4A–C, Supporting Information). FMT also reduced serum levels of IL‐1β, IL‐6, and TNF‐α, while increasing levels of IL‐4, IL‐10, and IL‐22 (Figure S4D–I, Supporting Information), indicating a significant anti‐inflammatory effect of gut microbiota. Interestingly, FMT increased XANA levels in both serum and colon, and activated the AhR (Figure 5W–Z). Furthermore, the beneficial effects of *AKK* supplementation were confirmed in DSS‐treated mice, where *AKK* supplementation was found to improve colitis and elevate XANA levels (Figure S4J–P, Supporting Information).

### 2.6 XANA is a Key Active Microbial Tryptophan Metabolite that Improves Colitis by Activating AhR

**Figure 6:**
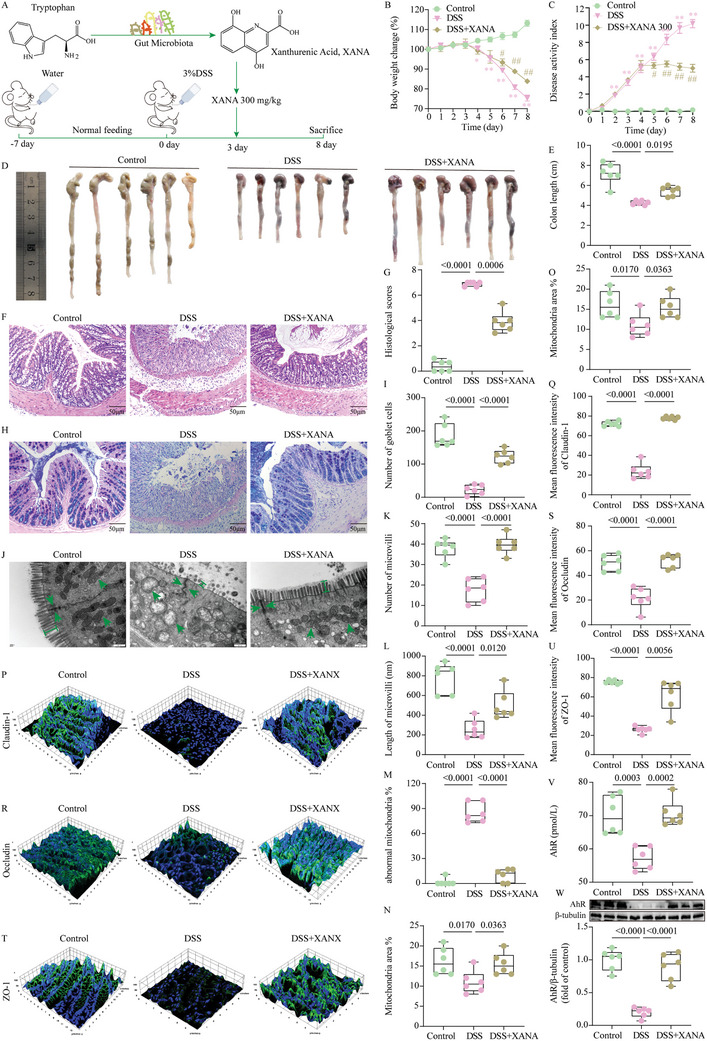
XANA is a key active microbial tryptophan metabolite that improves colitis by activating AhR. A) Schematic of the animal experiment 3. B) Body weight change (%) of mice (*n* = 6). C) DAI score (*n* = 6). D) Representative images of colonic tissue. E) Colon length (*n* = 6). F) Representative images of colonic sections stained with H&E (scale bars = 50 µm). G) Histological scores (*n* = 6). H) Representative images of colonic sections stained with AB‐PAS (scale bars = 50 µm). I) Number of goblet cells (*n* = 6). J) Representative TEM images of colonic tissue (scale bars = 500 nm). K) Number of microvilli (*n* = 6). L) Length of microvilli (nm, *n* = 6). M) % of abnormal mitochondria (*n* = 6). N) Mitochondria (µm^2^, *n* = 6). O) % of Mitochondria area (*n* = 6). P) IF imaging of tight junction structures using an antibody against Claudin‐1. Q) Mean fluorescence intensity of Claudin‐1 (*n* = 6). R) IF imaging of tight junction structures using an antibody against Occludin. S) Mean fluorescence intensity of Occludin (*n* = 6). T) IF imaging of tight junction structures using an antibody against ZO‐1. U) Mean fluorescence intensity of ZO‐1 (*n* = 6). V) ELISA analysis of AhR (*n* = 6). W) WB analysis of AhR (*n* = 6). ^*^ *P* < 0.05, ^**^ *P* < 0.01 versus control group; ^##^ *P* < 0.01 versus DSS group.

An increasing number of studies have revealed that the dysregulation of Trp metabolism regulated by intestinal microorganisms plays a role in the pathogenesis of diseases.^[^ ^10^ ^]^ XANA, a product of Trp metabolism, was identified as a key metabolite involved in the therapeutic effects of TLB on UC disease progression. This hypothesis was based on transcriptomics (screening for target genes), microbiomics (identifying differential bacterial populations), and metabolomics (highlighting critical differential metabolites). Consequently, we designed the following experiments to investigate this hypothesis (**Figure** **6**A). The results showed that, compared to the control group, the DSS group exhibited progressive weight loss, increased DAI scores, and shortened colons. However, XANA treatment significantly reversed these effects (Figure 6B–E). Additionally, XANA treatment notably restored the number of colonic crypts and goblet cells, reversing DSS‐induced colonic pathological damage (Figure 6F–I). XANA also reduced serum levels of IL‐1β, IL‐6, and TNF‐α, while increasing levels of IL‐4, IL‐10, and IL‐22 (Figure S5A–F, Supporting Information). TEM analysis revealed that XANA intervention restored the number and length of intestinal microvilli, inhibited mitochondrial damage, and improved tight junctions compared to the DSS group (Figure 6J–O). Furthermore, IF staining indicated that XANA treatment upregulated the levels of intestinal tight junction proteins (Figure 6P–U; and Figure S5G–I, Supporting Information). Importantly, AhR levels, which were significantly reduced in the DSS group compared to the control group, were notably restored following XANA treatment (Figure 6V,W). Interestingly, we found that XANA could directly bind to AhR by molecular docking, which was further verified by molecular dynamics simulation (Figure S5J–P, Supporting Information).

### 2.7 AhR is a Key Target that Mediates the Anti‐UC Effect of TLB

**Figure 7:**
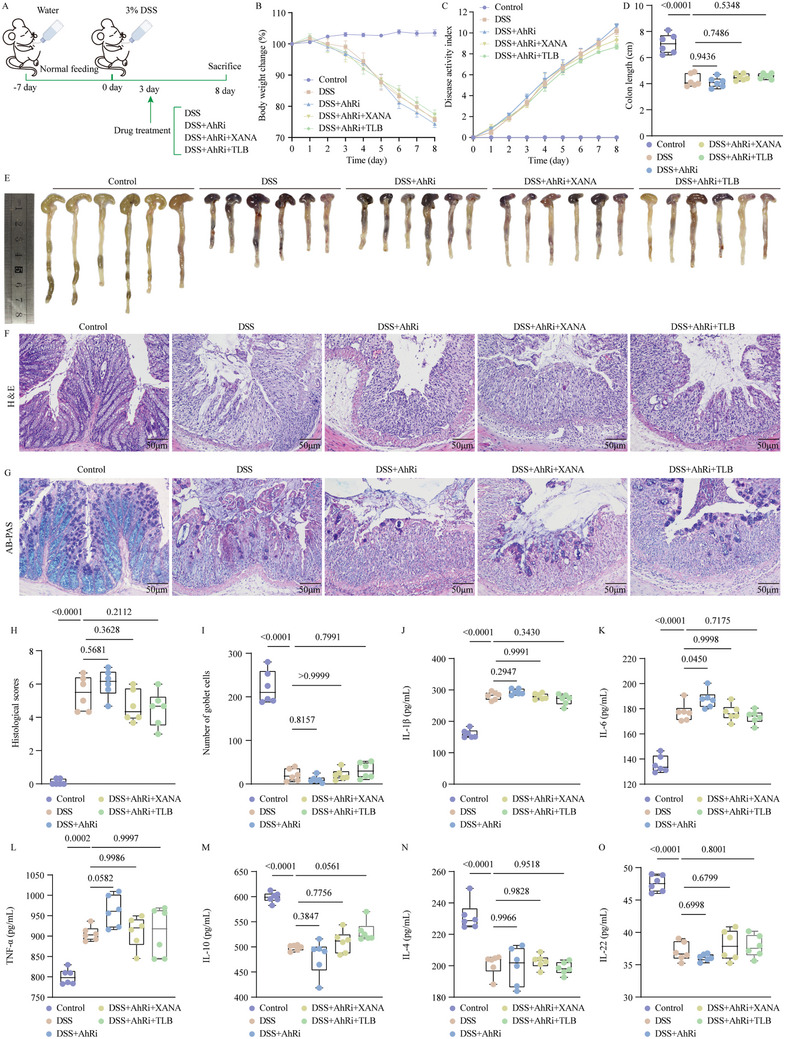
AhR is a key target that mediates the anticolitis effect of TLB. A) Schematic of the animal experiment 4. B) Body weight change (%) of mice. C) DAI score. D) Colon length. E) Representative images of the colonic tissue. F) Representative images of colonic sections stained with H&E (scale bars = 50 µm). G) Representative images of colonic sections stained with AB‐PAS (scale bars = 50 µm). H) Histological scores. I) Number of goblet cells. J) IL‐1β. K) IL‐6. L) TNF‐α. M) IL‐10. N) IL‐4. O) IL‐22.

Previous studies have demonstrated that indole and its derivatives, which are generated through microbial regulation of Trp metabolism, can prevent colitis by binding and activating AhR as ligands,^[^ ^17^ ^]^ indicating that TLB may ameliorate colitis by enriching the gut flora that metabolize Trp and its metabolite XANA to trigger AhR activation. To confirm this hypothesis, we exposed DSS‐treated mice to an AhR inhibitor to verify the contribution of AhR to the anti‐UC efficacy of TLB. AhR antagonist completely abolished the ameliorating effects of XANA on colitis, including body weight, DAI, colon length, colon histology, and serum levels of IL‐1β, IL‐6, TNF‐α, IL‐4, IL‐10, and IL‐22 (**Figure** **7**A–O). The protective effects of TLB therapy on body weight, DAI, colon length, and other markers were significantly attenuated by AhR inhibitors (Figure 7A–O).

## 3 Discussion

**Figure 8:**
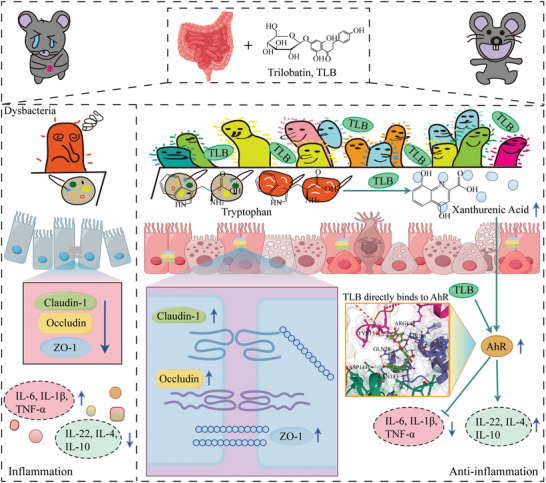
TLB alleviates DSS‐induced ulcerative colitis via AhR activation and xanthurenic acid production. UC is associated with dysbiosis, metabolic disruptions, and compromised intestinal barrier function. TLB restores microbial balance, enhancing Trp metabolism and producing XANA to activate AhR. TLB can also directly stimulate AhR, exerting anti‐inflammatory effects within the gastrointestinal tract.

This study demonstrates that TLB significantly alleviates intestinal inflammation in UC mouse models, as evidenced by reductions in the DAI, restoration of colon length, and improved intestinal barrier integrity. Additionally, TLB modifies the gut microbiota in UC mice, promoting the production of the Trp‐derived microbial metabolite XANA. Subsequent experiments demonstrated that XANA effectively restored the levels of tight junction proteins and mitigated intestinal inflammation in mice with ulcerative colitis. This anti‐inflammatory effect occurs through activation of the AhR pathway by TLB and XANA. In summary, TLB modulates gut microbiota to enhance XANA production, thereby activating AhR, improving intestinal barrier integrity, and ultimately reducing UC symptoms (**Figure** **8**). These findings not only confirm TLB's anti‐UC properties but also highlight its potential as a prebiotic agent with dual benefits of microbiota modulation and anti‐inflammatory efficacy. Given its demonstrated safety, efficacy, and ability to positively influence gut health, TLB emerges as a promising candidate for UC therapy.

Natural product‐based therapies have long been a cornerstone of traditional medicine, offering promising approaches to various health conditions.^[^ ^18^ ^]^ TLB, a natural sweetener derived from the plant *Lithocarpus polystachyus*, is known for its diverse biological activities.^[^ ^19^ ^]^ Previous research has demonstrated TLB's significant anti‐inflammatory properties, including its ability to inhibit neuroinflammation.^[^ ^14^, ^20^ ^]^ Additionally, TLB has been shown to improve cognitive dysfunction in senescence‐accelerated mice by targeting SIRT2 and restoring gut microbiota balance, thus influencing the gut‐brain axis.^[^ ^15a^ ^]^ Given these potent anti‐inflammatory effects, we investigated TLB's protective role against UC and the underlying mechanisms. Although the exact cause of UC remains unclear, growing evidence suggests that gut microbiota dysbiosis plays a critical role in its development.^[^ ^21^ ^]^ Studies have reported significant differences in gut microbiota diversity between UC patients and healthy individuals, particularly noting an increase in certain opportunistic pathogens and a decrease in anti‐inflammatory bacteria in UC.^[^ ^22^ ^]^ In our study, mice treated with DSS exhibited significant alterations in their gut microbiota, including reductions in beneficial bacteria like *AKK*, *Bacteroides*, and *Parabacteroides*, and increases in potentially harmful bacteria such as *Muribaculaceae*, *Clostridia*, *Lachnospiraceae*, and *Escherichia‐Shigella*. However, TLB treatment reversed these changes, increasing the abundance of beneficial bacteria while reducing pathogenic bacteria. Notably, *AKK*, *Muribaculum*, and *Alloprevotella* were negatively correlated with proinflammatory cytokines and positively correlated with anti‐inflammatory cytokines. Further functional analysis revealed that TLB‐modulated gut microbiota primarily influences metabolic pathways related to colitis, particularly Trp metabolism, sulfur metabolism, and glyoxylate and dicarboxylate metabolism.

Trp is an essential amino acid that can only be obtained from dietary sources. Gut microbiota plays an important role in Trp metabolism. Gut microbes can directly convert Trp into various molecules, such as indole and its derivatives, to maintain intestinal homeostasis by regulating the expression of proinflammatory and anti‐inflammatory cytokines.^[^ ^23^ ^]^ Our metabolomics analysis confirmed a significant increase in XANA levels following TLB treatment. Indole derivatives, including XANA, are recognized as ligands of the AhR, a crucial regulator of gut immunity and a key player in initiating downstream signaling pathways.^[^ ^11^, ^24^ ^]^ Furthermore, our investigations revealed a significant activation of AhR through transcriptomic analyses, ELISA, and Western blotting techniques. AhR activation has been linked to the alleviation of colitis by reducing inflammatory cytokines like IL‐1β, IL‐6, and TNF‐α, while enhancing anti‐inflammatory cytokines, such as IL‐4, IL‐10, and IL‐22, and strengthening the intestinal epithelial barrier. Our results suggest that TLB promotes XANA production through Trp metabolism regulated by the gut microbiota. More importantly, we identified a key role of AKK in promoting the upregulation of the tryptophan metabolite XANA. Consistent with this, both TLB and XANA treatments significantly increased AhR protein levels in the colon, reduced inflammatory cytokines, elevated anti‐inflammatory cytokines in serum, and repaired intestinal barrier function. Notably, SPR and MST experiments have provided direct evidence of a physical interaction between TLB and AhR. These findings, combined with observed changes in downstream inflammatory mediators and anti‐inflammatory cytokines, indicate that TLB can act as an AhR ligand, directly activating its function. This molecular interaction supports the role of TLB as an AhR agonist. Activation of AhR by TLB not only suppresses intestinal inflammation but also promotes gut homeostasis and reshapes gut bacterial communities. Additionally, through modulating gut microbiota and their metabolites, TLB‐mediated AhR activation further enhances AhR expression, rescuing intestinal epithelial cells and fostering a positive feedback loop. These findings highlight the dual roles of TLB in both directly binding to AhR and indirectly modulating its expression and activity through microbiota interactions. In summary, our findings support the existence of a “TLB‐gut microbiota‐XANA‐AhR” regulatory axis, highlighting TLB's potential as a therapeutic agent for UC through its modulation of gut microbiota and subsequent activation of AhR. Notably, AhR antagonists eliminated the beneficial effects of XANA on colitis, but only attenuated the effects of TLB. Given this difference, we speculate that TLB's ability to relieve colitis may be due to a variety of mechanisms. For example, we found in transcriptomics that TLB can also significantly inhibit TNF signaling, suggesting that TNF signaling may also be involved in the anticolitis effect of TLB.

While this study provides compelling evidence that gut microbiota and its metabolite XANA are central to TLB's anticolitis effects, there are limitations. XANA is one of the most important metabolite whose efficacy has been validated through treatment with XANA; however, the functions of the other metabolites regulated by TLB remain unclear. In addition XANA is an indole metabolite produced by bacterial fermentation of Trp, suggesting that multiple bacterial species may contribute to its production. Although our study demonstrated TLB's regulatory effects on the microbiota, further research is required to identify other bacteria capable of producing indole derivatives like XANA. This will be essential for comprehensively understanding TLB's anticolitis mechanisms and advancing its development as a therapeutic agent.

## 4 Experimental Section

### Animals and UC Model

Male C57BL/6J mice (20–23 g; 8–10 weeks) were purchased from Hunan SJA Laboratory Animal Co., Ltd. (SCXK [XIANG] 2019‐0004). Mice were housed five per cage in soft wood‐shaving‐lined plastic cages within a specific pathogen‐free facility (SYXK [QIAN] 2021‐0003), maintained at 22–24 °C, 35%–55% humidity, and a 12‐h light/dark cycle. Animal experiments strictly adhered to the regulations of the Ethics Committee for Animal Experiments at Zunyi Medical University (ZMU21‐2203‐538). Dextran Sulfate Sodium Salt (DSS, Cat No.160110, MP Biomedicals), a polyanionic derivative of dextran, was dissolved in drinking water at a concentration of 3% to induce UC. DSS (molecular mass 36–50 kDa) is one of the most common and effective chemical inducers for modeling UC in animals.^[^ ^25^ ^]^

### Treatments and Sample Collection

Experiment 1: To evaluate the efficacy of TLB and SASP in UC mitigation, mice were divided into seven groups (*n* = 9): control, control+TLB (purity >99%) 40 mg kg^−1^, DSS, DSS+TLB (10, 20, 40 mg kg^−1^), and DSS+sulfasalazine (SASP, 200 mg kg^−1^, MedChemExpress). The experimental workflow was depicted in Figure 1A. After a 1‐week acclimatization period, the mice were allowed to drink water containing 3% w/v DSS for 8 days to induce UC, with the DSS solution being replaced every 3 days to maintain efficacy. Three days post‐DSS induction, the mice were administered with TLB, SASP, or an equivalent volume of saline via gavage once daily for 5 consecutive days. From day 1 to day 8, body weight, stool consistency, and rectal bleeding were recorded daily between 14:00 and 16:00. On day 8, fresh fecal samples were aseptically collected for 16S rRNA analysis. Blood was then collected from the retro‐orbital sinus under anesthesia, and serum was isolated and stored at −80 °C for subsequent experiments. All animals were euthanized by cervical dislocation, and the colons were promptly excised, rinsed with PBS, and measured for length. Colon tissues were collected for further analysis, with a small portion of the mid‐colon sectioned for histological staining, while the remaining tissue was frozen at −80 °C for subsequent experiments.

Experiment 2: To explore the microbial mechanisms behind TLB's UC‐ameliorative effects, a gut microbiota depletion approach was employed using an antibiotic cocktail (ABX), as illustrated in Figure 4A. After a 1‐week adaptive feeding period, all mice received ABX in their drinking water for 14 days to generate pseudogerm‐free conditions. Mice were then randomly assigned into three groups (*n* = 9): vehicle, DSS, and DSS+FMT. To induce UC, DSS was administered over 8 days, concurrent with continued ABX treatment to sustain microbiota depletion. Fecal samples were collected throughout the experiment. From day 3 of DSS treatment, FMT were administered via gavage (0.2 mL feces suspension) daily for 5 days. Body weight, stool consistency, and rectal bleeding were monitored daily. At the end of the experiment, blood and colon samples were collected as previously described.

Experiment 3: To verify the role of metabolites in improving UC damage, mice were randomly divided into 3 groups (*n* = 6): control, DSS, and DSS+XANA (300 mg kg^−1^, TargetMol Chemicals Inc.). The experimental workflow was depicted in Figure 6A. After a 1‐week acclimatization period, the mice were allowed to drink water containing 3% w/v DSS for 8 days to induce UC, with the DSS solution replaced every 3 days to maintain efficacy. Starting from day 3, XANA 300 mg kg^−1^ was administered intragastric. Body weight, stool consistency, and rectal bleeding were recorded daily. At the end of the experiment, blood and colon tissues were collected as previously described.

Experiment 4: To investigate the pivotal role of AhR in the amelioration of UC by TLB, mice were administered an AhR inhibitor (AhRi), Stemregenin 1.^[^ ^26^ ^]^ The experimental workflow was depicted in Figure 7A. Following a 7‐day acclimatization period, the mice were randomly assigned to five groups: control, DSS, DSS+AhRi, DSS+AhRi+XANA, and DSS+AhRi+TLB. The mice had unrestricted access to drinking water containing 3% DSS (which was refreshed or replenished every 3 days), while TLB at a dosage of 40 mg kg^−1^ or XANA at 300 mg kg^−1^ was administered on day 3. Concurrently, StemRegenin 1 (50 mg kg^−1^; MedChemExpress) was given in the AhRi group. Mice underwent continuous gavage for 5 days with samples collected on the eighth day. Throughout the experiment, fecal occult blood levels were assessed daily by weighing.

### Assessment of Disease Activity Index (DAI)

The DAI assesses the severity of disease by recording weight loss, stool consistency, and the presence of blood in the stool.^[^ ^27^ ^]^ During the UC induction period, the DAI of mice was recorded daily. The weight loss scoring criteria were as follows: 0) <2% loss, 1) 2%–5% loss, 2) 5%–10% loss, 3) 10%–15% loss, 4) and ≥15% loss. Stool consistency was scored as follows: 0) normal; 1) slightly soft stool; 2) loose stool; 3) watery stool. Fecal blood was assessed using the Fecal Occult Blood Test Kit (o‐tolidine method, BC8270, Solarbio) according to the manufacturer's instructions. The scoring criteria for fecal blood were: 0) no color change within 2 min; 1) light green to green; 2) light green to blue–brown; 3) immediate blue–brown turning to black–brown; 4) immediate black–brown.

### Histopathological Assessment

Colon tissues were fixed in 4% paraformaldehyde (P1110, Solarbio) for 48 h, dehydrated (TP1020, Leica), and embedded in paraffin for sectioning at 4 µm (BG‐1150, ROM‐2245, Leica).^[^ ^28^ ^]^ Routine hematoxylin and eosin (H&E) staining was performed using an automated stainer (ST5010, Leica Autostainer XL). The stained slides were digitally scanned at 40 × magnification using the Teksqray SQS‐1000 slide scanning imaging system, allowing for visualization and analysis of the tissue sections. Histological scoring was conducted based on a previously described scoring system, utilizing two histological parameters to assess the severity of DSS‐induced UC.^[^ ^29^ ^]^ The criteria for scoring tissue inflammation were as follows: no significant increase in the lamina propria (0 points); mild increase in the lamina propria (1 point); moderate expansion of the lamina propria with increased infiltration of inflammatory cells (2 points); marked increase in the lamina propria size with extensive nuclear infiltration into surrounding areas (3 points). The criteria for scoring crypt damage were: intact crypts extending down to the muscularis mucosae (0 points); crypt base shortened and missing by 1/3 (1 point); crypt base missing by 2/3 with focal epithelial thinning (2 points); complete loss of crypts with the surface epithelium remaining (3 points); complete loss of crypts with surface erosion (4 points). Tissue sections were analyzed by three experienced scientists blinded to the groupings. Histopathological scores were established based on these criteria and averaged to reflect the extent of DSS‐induced colonic damage or inflammation.

### Alcian Blue‐Periodic Acid‐Schiff (AB‐PAS) Staining

After deparaffinization and rehydration of colon tissue sections, AB‐PAS staining was performed according to the instructions provided in the manual (G1285, Solarbio). Briefly, slides were immersed in Alcian blue staining solution for 10 min, followed by rinsing three times with distilled water. Then, slides were oxidized for 5 min using the oxidizing agent, followed by staining with Schiff's reagent for 10 min. Hematoxylin staining for 1 min was used to stain cell nuclei, followed by differentiation in Scott's bluing solution for 3 min. Dehydration and mounting in a transparent sealant were then performed for examination under a light microscope (BX 43 Olympus, Tokyo, Japan). Goblet cells were quantified using imagej.js (v0.5.7; https://ij.imjoy.io).

### Assay of Inflammatory Indexes

The inflammatory response was quantified through Enzyme‐linked immunosorbent assay (ELISA) of inflammatory cytokines in serum. Briefly, mice were anesthetized, blood was collected via eyeball extraction, allowed to stand for 4 h, and then serum was collected by centrifugation at 4 °C (10 000 × g, 20 min). Subsequently, levels of IL‐1β (RJ16944, Renjiebio), IL‐6 (RJ16958, Renjiebio), TNF‐α (RJ17929, Renjiebio), IL‐4 (RJ16956, Renjiebio), IL‐10 (RJ16932, Renjiebio), and IL‐22 (RJ16948, Renjiebio) were determined using commercial ELISA kits according to the manufacturer's instructions.

### RNA‐Seq Analyses

Total RNA was extracted from colonic samples of the DSS and DSS+TLB 40 mg kg^−1^ groups using Trizol buffer. The integrity, quality, and purity of the RNA samples were assessed. Qualified RNA was sequenced on the BGISEQ‐500RS RNA‐Seq platform. Gene expression was calculated using fragments per kilobase of transcript per million mapped reads (FPKM) for each sample. In this study, genes with fold change (FC) > 1.5 and *Q* value < 0.05 were identified as differentially expressed genes (DEGs). Volcano plots, heatmaps, Gene Ontology (GO) analysis, and Kyoto Encyclopedia of Genes and Genomes (KEGG) analysis were generated using OmicStudio tools (https://www.omicstudio.cn/tool).^[^ ^30^ ^]^

### Molecular Docking

Briefly, the human AhR X‐ray crystal structure was obtained from the Protein Data Bank (PDB ID: 5NJ8) for molecular docking. As mentioned earlier, Autodock Vina 1.2.0 is used to predict small molecular–protein interactions by molecular docking TLB and XANA with AhR, respectively.^[^ ^31^ ^]^ Subsequently, the best binding conformations were imported into LigPlot^+^ (v.2.2.8) for analysis, and the ligand–protein complex was visualized using PyMOL protein visualization software.^[^ ^32^ ^]^

### Molecular Dynamics (MD) Simulation

Protein and small molecule structure files were imported into Gromacs 2022.3.^[^ ^33^ ^]^ Topology files and the simulation box were generated using the pdb2gmx and gmx editconf commands, respectively. The MD simulation system underwent energy minimization using the steepest descent method. Simulations were conducted under isothermal conditions at a static temperature of 300 K and constant pressure (1 Bar), using the Amber99sb‐ildn force field. Subsequently, a 100 000‐step equilibration was performed under isothermal‐isovolumetric ensemble (NVT) conditions, followed by a 100 000‐step equilibration under isothermal‐isobaric ensemble (NPT) conditions. Both equilibrations were conducted with a coupling constant of 0.1 ps and a duration of 100 ps. The gmx grompp and gmx mdrun commands were used to carry out MD simulations for the protein and small molecule over a duration of 100 ns, during which conformational information was recorded. The built‐in tools of Gromacs were employed to analyze the trajectory, calculating data such as root mean square deviation (RMSD), root mean square fluctuation (RMSF) for each amino acid, and the free energy landscape.

### Microscale Thermophoresis (MST)

The binding affinity of TLB with AhR were measured by MST. Briefly, The His‐tagged recombinant human AhR (Cloud‐Clone Corp., China) was labeled with the Monolith His‐Tag Labelling Kit RED‐tris‐NTA 2nd Generation (NanoTemper Technologies). Then, the samples were incubated for 1 h at room temperature and analyzed by a Monolith NT.115 MST device (NanoTemper, Germany). Using nanoblue excitation, the LED light was adjusted to 100% excitation power, and MST power was set to 40%. The *K*_D_ values were determined using the MO.AffinityAnalysis software.

### Surface Plasmon Resonance (SPR) Analysis

The binding affinity of TLB to recombinant human AhR was determined using Biacore 1 K instrument equipped with sensor chip CM5 (Cytiva, Sweden). Briefly, AhR protein in acetate 4.0 were immobilized on the sensor after activation by 100 µL EDC and 100 µL NHS in water solution. Different concentrations of TLB, including 100, 50, 25, 12.5, 6.25, 3.13, and 1.56 µm were prepared with running buffer (phosphate‐buffered saline with Tween‐20). Data processing and analysis were performed using Biacore evaluation software.

### AhR Activity Determination

The levels of AhR in colon tissues were detected using Western blot and ELISA. Briefly, protein concentration was determined using a BCA protein assay kit. Twenty micrograms of total protein from colon tissues were separated by SDS‐PAGE and then transferred onto a 0.22 µm polyvinylidene fluoride (PVDF) membrane (Millipore, MA). After blocking with 5% nonfat milk at room temperature for 2 h, the membrane was incubated overnight at 4 °C with an AhR polyclonal antibody (1:50, 28727‐1‐AP, Proteintech). The membrane was then incubated with horseradish peroxidase (HRP)‐conjugated antimouse or antirabbit IgG at room temperature for 1 h. Proteins were visualized using enhanced chemiluminescence HRP substrate and quantified using imagej.js (v0.5.7; https://ij.imjoy.io). Additionally, the AhR levels in colon tissues were measured using an ELISA kit according to the manufacturer's instructions (RJ17093, Renjiebio).

### XANA Assay

The XANA ELISA kit (SUJX98053, Renjiebio) was employed to detect XANA in serum and colon tissue. Serum or colon tissue, standard products, and HRP‐labeled competing antigens were added to the micropores precoated with xanthuric acid antigen, followed by warming and thorough washing. The substrate TMB was utilized for color development, and the depth of the color was negatively correlated with the xanthurenic acid in the sample. The absorbance (OD value) was measured by an enzyme‐labeled instrument at a wavelength of 450 nm, and the sample concentration was calculated.

### Transmission Electron Microscopy (TEM) Analysis

The ultrastructures of gut microbiota, colonic microvilli, tight junctions, and mitochondria were observed using TEM. Briefly, mouse colonic tissues were fixed in 3% glutaraldehyde at 4 °C for 24 h and subsequently fixed in 1% osmium tetroxide. After dehydration, infiltration, and embedding, ultrathin sections (≈60 nm) were prepared using an ultramicrotome (UC7rt, Leica). The sections were mounted onto copper grids and imaged using a transmission electron microscope (JEM‐1400FLASH, JEOL Ltd.). The colonic microvilli and mitochondria were then quantified using ImageJ software (v0.5.7; https://ij.imjoy.io).

### Immunofluorescent (IF) Staining

IF staining was employed to detect the levels of tight junction‐related proteins. Briefly, tissue sections were deparaffinized and then incubated overnight at 4 °C with primary antibodies, including Claudin 1 (1:100, ab307692, Abcam), Occludin (1:100, ab216327, Abcam), and ZO‐1 (1:100, 21773‐1‐AP, Proteintech). After primary antibody incubation, sections were incubated with the corresponding secondary antibody, CoraLite488 (1:500, SA0013‐2, Proteintech), for 4 h. The slides were then mounted with an antifade mounting medium containing DAPI and observed using a confocal laser scanning microscope (STELLARIS 5, Leica). imagej.js (v0.5.7; https://ij.imjoy.io) was used for 3D surface plot and quantification of fluorescence intensity.

### 16S rRNA Sequencing

To investigate the impact of TLB on the gut microbiota of UC mice, fresh fecal samples from the control, DSS, and DSS+TLB 40 mg kg^−1^ groups were analyzed through 16S rRNA sequencing. Total microbial DNA was extracted from various microbiome samples using the cetyltrimethylammonium bromide method, followed by an assessment of the integrity, quality, and purity of the samples. The qualified DNA samples were sequenced using the NovaSeq 6000 platform. Furthermore, microbial diversity analysis was performed using the OmicStudio tool (https://www.omicstudio.cn/tool).

### Untargeted Metabolome

Serum samples were collected on ice, and metabolites were extracted with 80% methanol buffer. Samples were analyzed using an LC‐MS system. Chromatographic separations were performed on an UltiMate 3000 UPLC System (Thermo Fisher Scientific) with an ACQUITY UPLC T3 column (100 mm × 2.1 mm, 1.8 µm, Waters) at 40 °C. The mobile phase consisted of 5 mm ammonium acetate and 5 mm acetic acid (solvent A) and acetonitrile (solvent B). The flow rate was 0.3 mL min^−1^ with gradient elution: 0–0.8 min, 2% B; 0.8–2.8 min, 2%–70% B; 2.8–5.6 min, 70%–90% B; 5.6–6.4 min, 90%–100% B; 6.4–8.0 min, 100% B; 8.0–8.1 min, 100%–2% B; 8.1–10 min, 2% B. A high‐resolution tandem mass spectrometer Q‐Exactive (Thermo Scientific) detected metabolites in both positive and negative ion modes. Precursor spectra (70–1050 m/z) were collected at 70 000 resolutions with an AGC target of 3e6 and a maximum injection time of 100 ms. Fragment spectra were collected at 17 500 resolutions with an AGC target of 1e^5^ and a maximum injection time of 80 ms. A quality control sample (pooled from all samples) was analyzed every 10 samples to ensure LC‐MS stability. Student *t‐*tests identified differences in metabolite concentrations between phenotypes, with *P*‐values adjusted for multiple tests using FDR (Benjamini–Hochberg). Supervised PLS‐DA conducted through metaX was used to discriminate variables between groups, with a VIP cut‐off value of 1.0 to select important features. Differential ions were considered significant if they met the following criteria: ratio > 1.5 or ratio < 1/1.5, *Q* value < 0.05, and VIP > 1. Functional analysis of the differential ions was then performed using the OmicStudio tool (https://www.omicstudio.cn/tool).

### Analysis of Trp Metabolism

In brief, a 100 µL serum sample was added to 500 µL of cold ddH_2_O, and was swirled and ultrasonically treated for 10 min, respectively. After the supernatant was collected, 500 µL of ice methanol was added to the EP tube and extracted with the organic phase for LC‐MS/MS analysis. The separation and quantification of target compounds were performed using an AB sciex jasper ultra performance liquid chromatograph coupled with an AB SCIEX 4500MD triple quadrupole mass spectrometer. The chromatographic separation was carried out on an Agilent Poroshell 120 EC‐C18 (3.0 × 150 mm, 2.7 µm) column. The mobile phase consisted of A) ammonium acetate aqueous solution (5 mmol L^−1^) with 0.01% formic acid and B) acetonitrile were used as mobile phases. Injection volume: 5 µL. Temperature: 40 °C. AB SCIEX 4500MD triple quadrupole mass spectrometer, equipped with an ESI Turbo Ion‐Spray interface, operating in both positive and negative ion modes. The ESI source operation parameters were as follows: ion source, turbo spray; Temperature: 400 °C; Ion Spray Voltage: 4500 V(Positive), −4500 V (Negative); Curtain Gas: 30.0 psi; CE for individual MRM transitions was done with further CE optimization. A specific set of MRM transitions were monitored for each period. BoxPlot was performed using the OmicStudio tools at https://www.omicstudio.cn/tool.

### Fecal Microbiota Transplantation

To determine the role of gut microbiota in alleviating UC, an FMT experiment was conducted. Specifically, all mice received an antibiotic mixture (ABX: Ampicillin [1 g L^−1^, CAS No.69‐53‐4, MedChemExpress], Vancomycin [1 g L^−1^, CAS No.1404‐90‐6, MedChemExpress], Neomycin sulfate [1 g L^−1^, CAS No. 1405‐10‐3, MedChemExpress], and Metronidazole [1 g L^−1^, CAS No. 443‐48‐1, MedChemExpress]) in their drinking water for 14 days to create pseudogerm‐free mice.^[^ ^34^ ^]^ The ABX water was replaced every 3 days to maintain efficacy. Fresh fecal samples (1 g) were collected daily from UC mice treated with TLB 40 mg kg^−1^ for 5 days (donor, *n* = 9), suspended in 5 mL of distilled water, centrifuged at 1000 rpm at 4 °C, and filtered through a 200‐mesh sieve to obtain fecal microbiota suspension.^[^ ^35^ ^]^ The germ‐free UC mice (recipient, *n* = 9) were treated with 0.2 mL of the fecal microbiota suspension for 5 consecutive days, starting 3 days after DSS induction.

### Bacteria Culture and Supplementation in UC Mice

To investigate the role of *AKK* in improving colitis and its relationship with XANA metabolites, AKK in DSS‐treated mice was supplemented. AKK (ATCC BAA‐835, MINGZHOU Bio) was cultured as previously described.^[^ ^36^ ^]^ Briefly, live AKK bacteria were harvested after repeated centrifugation and PBS washing. The collected bacteria were resuspended in sterile water to achieve a final concentration of 1 × 10^9^ CFU mL^−1^. From the third day of DSS treatment, AKK was administered via oral gavage (0.2 mL) daily for 5 consecutive days. Body weight, stool consistency, and rectal bleeding were monitored daily throughout the experiment. At the conclusion of the study, blood and colon samples were collected as previously outlined.

### Statistical Analysis

Values are expressed as s mean ± standard error of the mean (mean ± SEM) of six or nine independent experiments. In addition, three or more groups were compared using one‐way ANOVA followed by Bonferroni (same variance assumed) or Dunnett's T3 (equal variance not assumed) using SPSS version 29.0 (SPSS, Inc., Chicago, USA). A significance level of *P* < 0.05 was considered statistically significant.

## Conflict of Interest

The authors declare that the research was conducted in the absence of any commercial or financial relationships that could be construed as a potential conflict of interest.

## Supporting information



Supporting Information

## Data Availability

The data that support the findings of this study are available from the corresponding author upon reasonable request.
