# Supplementary material for: The Gut Microbiota‐Xanthurenic Acid‐Aromatic Hydrocarbon Receptor Axis Mediates the Anticolitic Effects of Trilobatin
Source: Adv Sci (Weinh). 2025 Jan 21;12(10):2412234. doi: 10.1002/advs.202412234 (PMC11904984; doi:10.1002/advs.202412234)
Supplement: Supplementary file 1 — Supporting Information [file ADVS-12-2412234-s001.docx]

Supporting Information

The Gut Microbiota-Xanthurenic Acid-Aromatic Hydrocarbon Receptor Axis Mediates Anti-Ulcerative Colitis Effect of Trilobatin

Xiaoyu Wu, Jiajia Wei, Wang Ran, Dongjing Liu, Yang Yi, Miaoxian Gong, Xin Liu, Qihai Gong, Haibo Li^*^, Jianmei Gao^*^


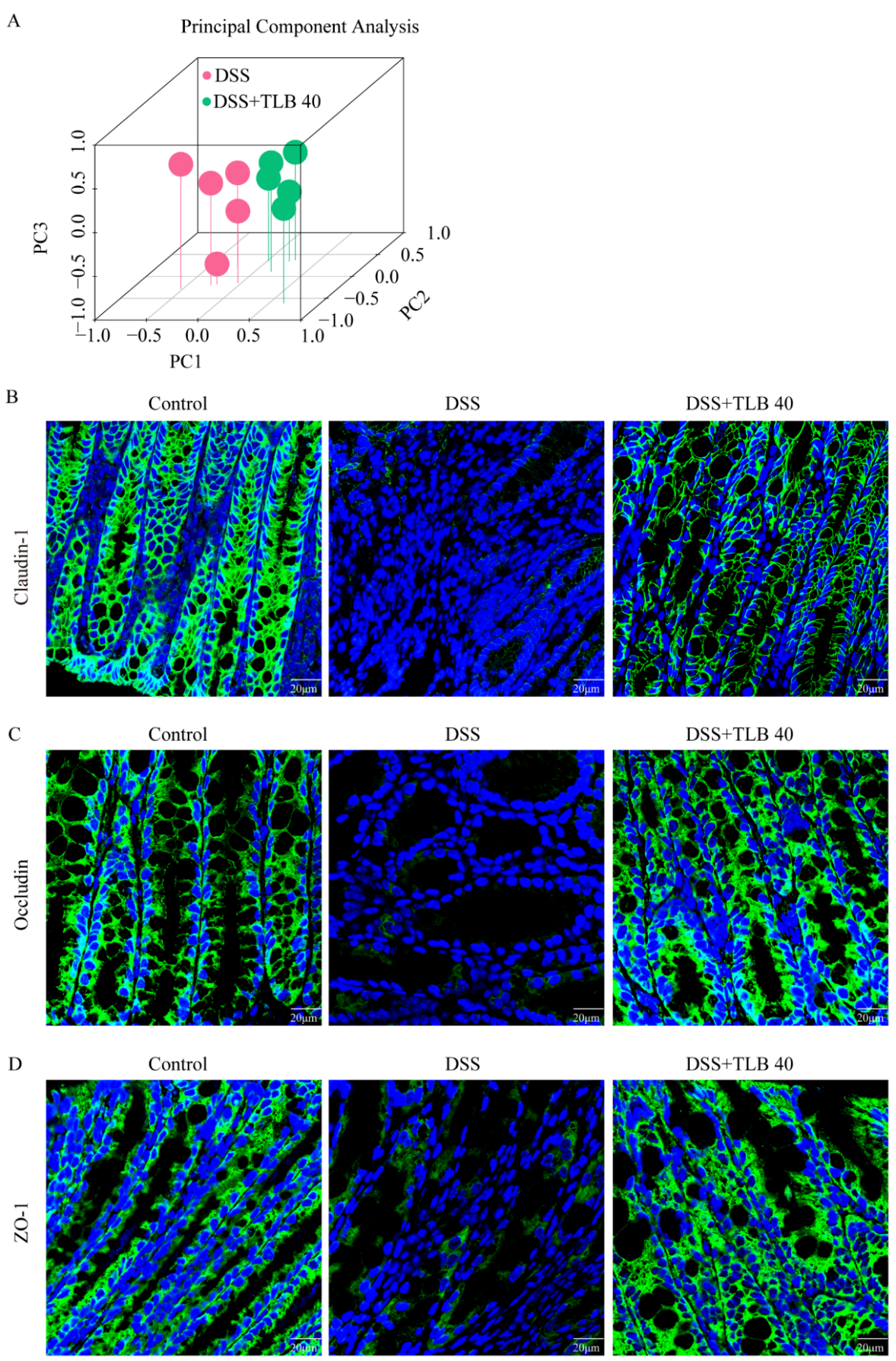


**Figure S1.** (A) PCA analysis for DSS+TLB 40 mg/kg *vs* DSS (n=5). (B) IF imaging (2D) of tight junction structures using an antibody against Claudin-1. (C) IF imaging (2D) of tight junction structures using an antibody against Occludin. (D) IF imaging (2D) of tight junction structures using an antibody against ZO-1.


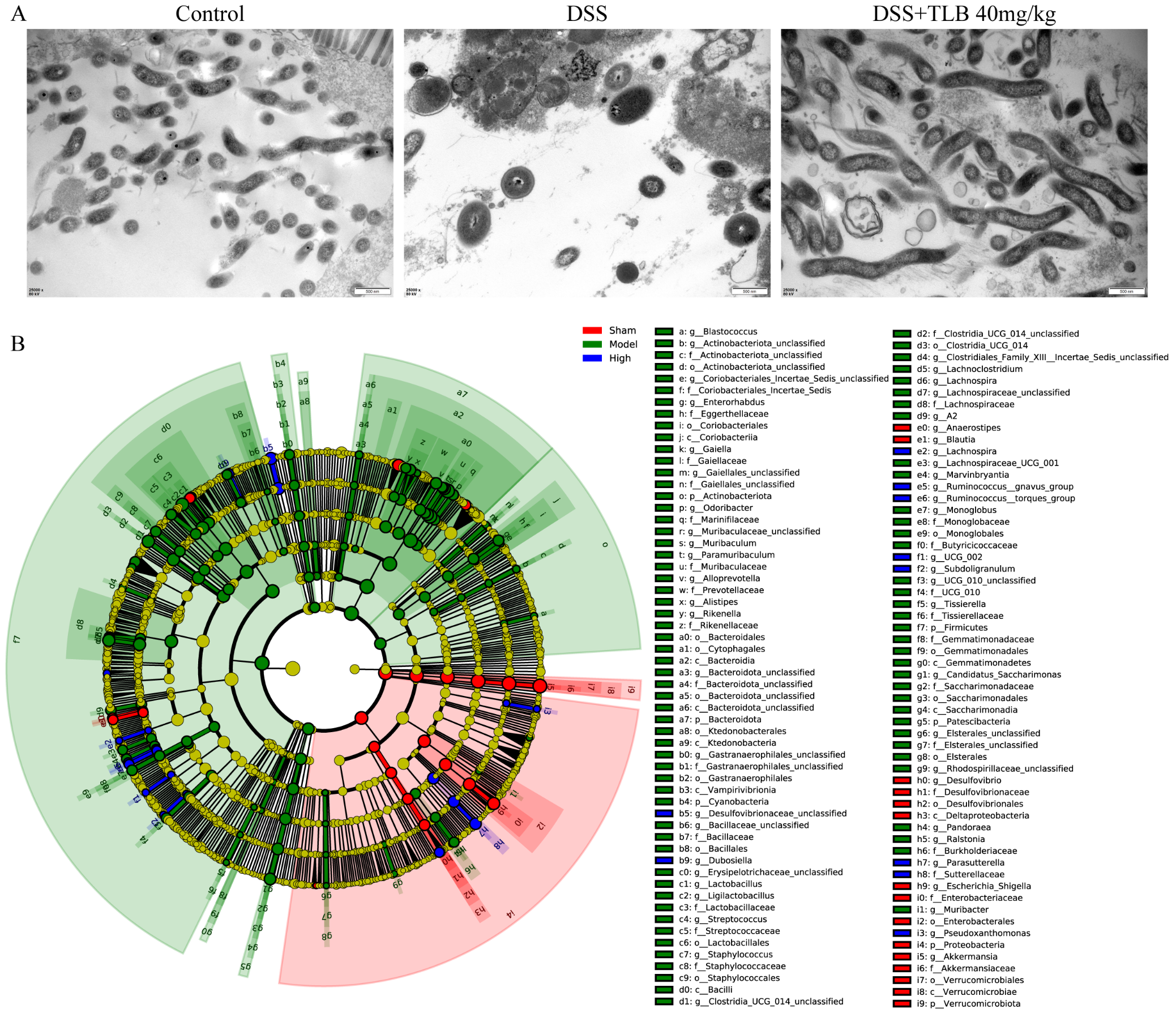


**Figure S2.** (A) Representative TEM images of gut microbiota (scale bars=500nm). (B) LEfSe analysis.


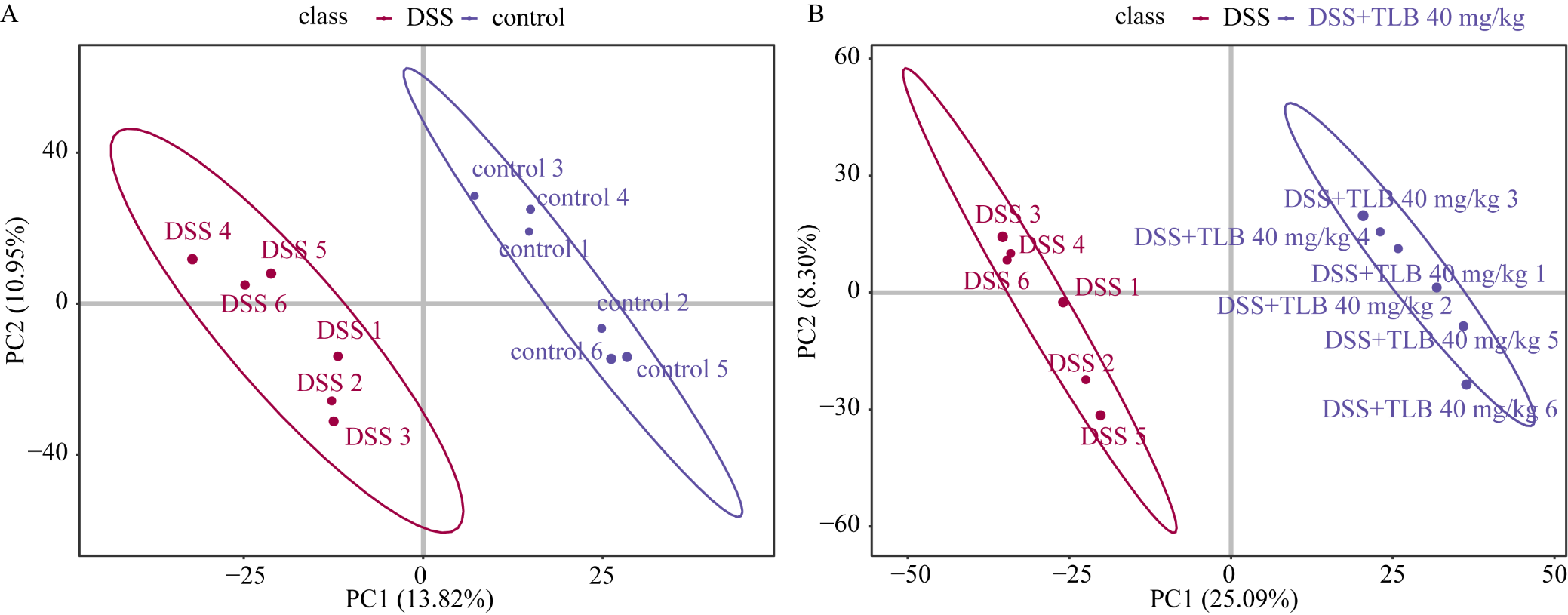


**Figure S3.** (A) PLS-DA analysis for DSS *vs* control (n=6). (B) PLS-DA analysis for DSS+TLB 40 mg/kg *vs* DSS (n=6).


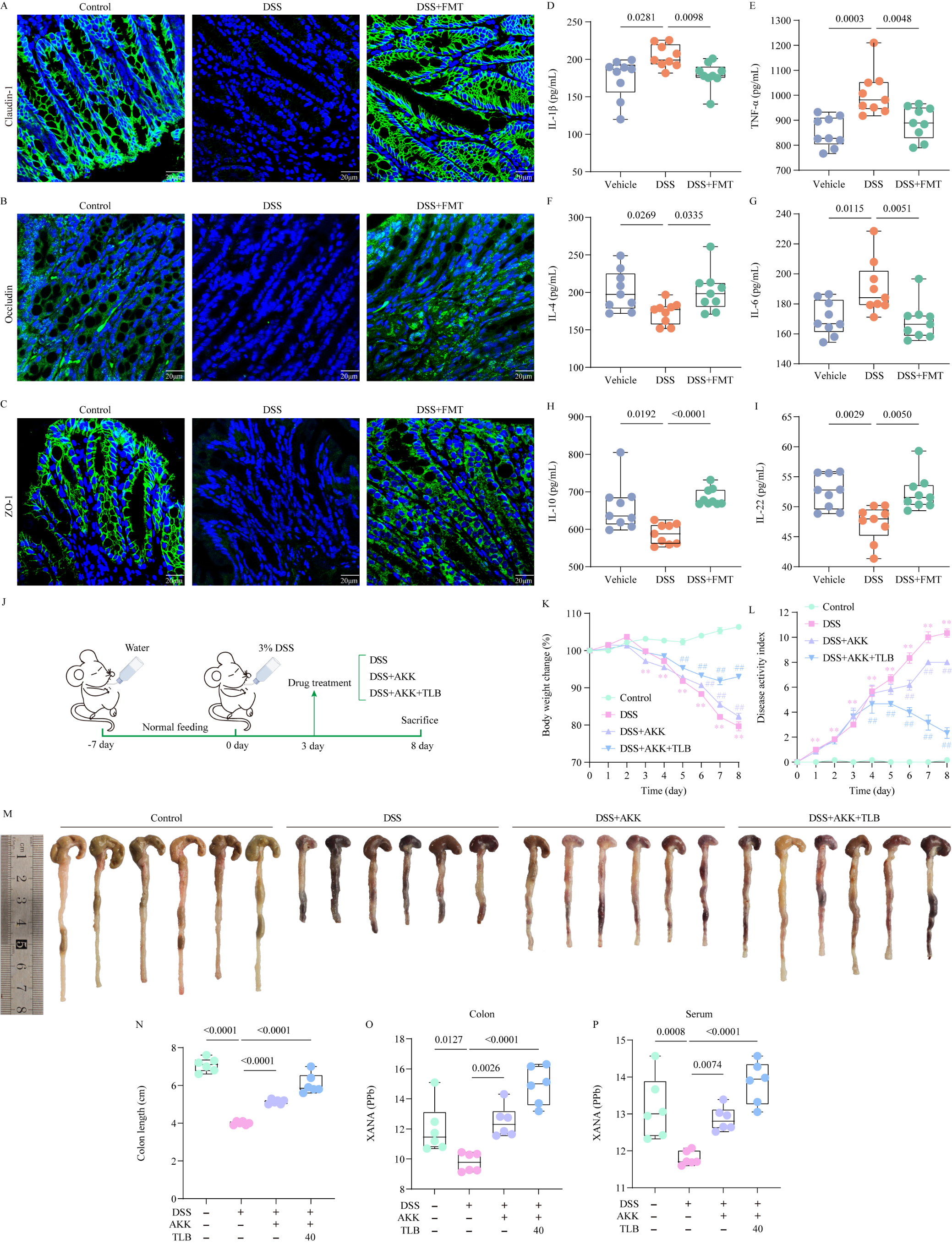


**Figure S4.** (A) IF imaging (2D) of tight junction structures using an antibody against Claudin-1. (B) IF imaging (2D) of tight junction structures using an antibody against Occludin. (C) IF imaging (2D) of tight junction structures using an antibody against ZO-1. (D) IL-1β (n=9). (E) TNF-α (n=9). (F) IL-4 (n=9). (G) IL-6 (n=9). (H) IL-10 (n=9). (I) IL-22 (n=9). (J) Schematic of experiment. (K) Body weight change (%) of mice. (L) DAI score. (M) Representative images of the colonic tissue. (N) Colon length. (O) ELISA analysis of XANA in colon (n=6). (P) ELISA analysis of XANA in serum (n=6). ^**^*P* < 0.01 versus control group; ^##^*P* < 0.01 versus DSS group.


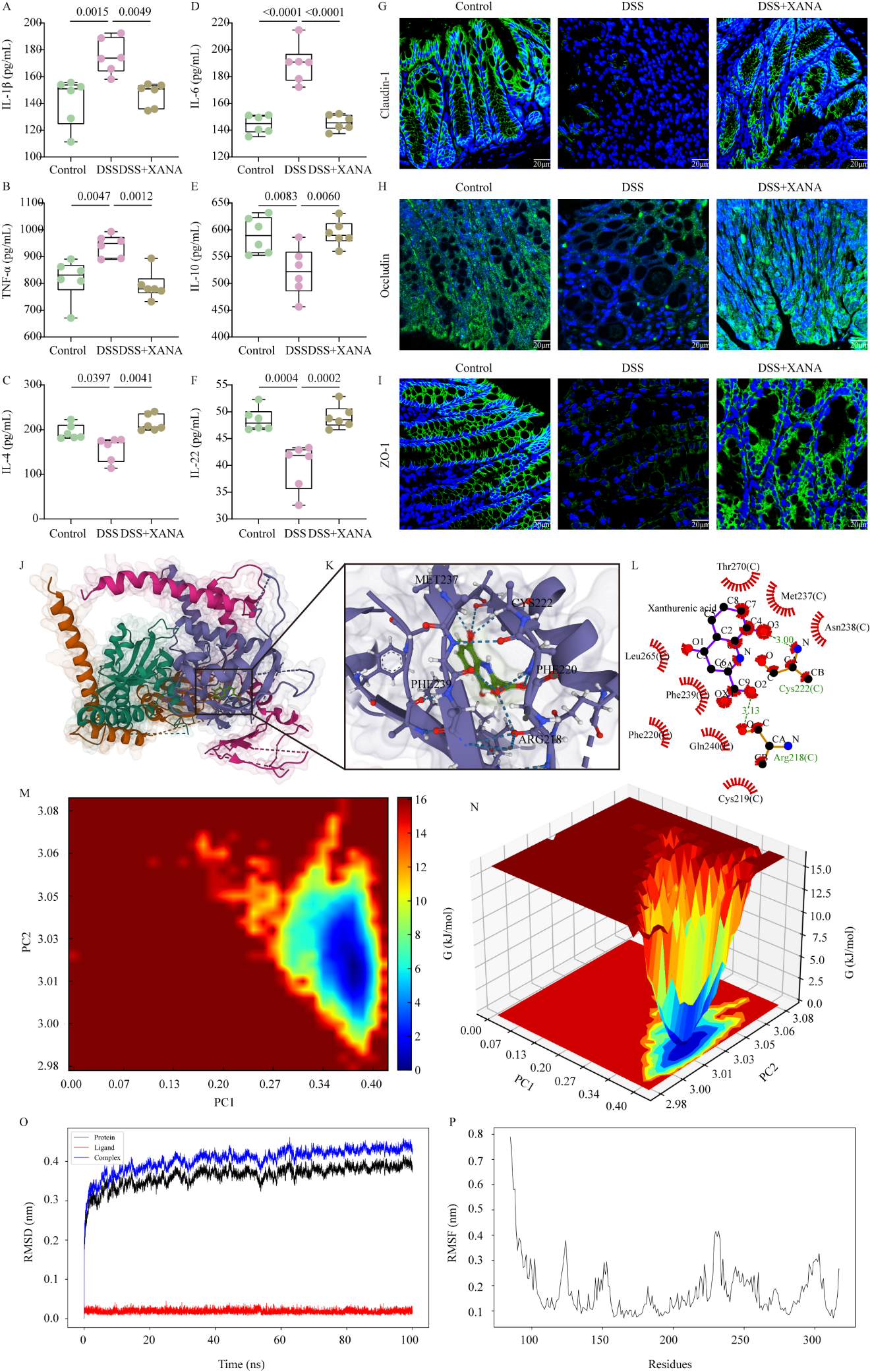


**Figure S5.** (A) IL-1β (n=6). (B) TNF-α (n=6). (C) IL-4 (n=6). (D) IL-6 (n=6). (E) IL-10 (n=6). (F) IL-22 (n=6). G. IF imaging (2D) of tight junction structures using an antibody against Claudin-1. (H) IF imaging (2D) of tight junction structures using an antibody against Occludin. (I) IF imaging (2D) of tight junction structures using an antibody against ZO-1. (J) The entire view of the binding sites between XANA onto AhR. (K) A close-up view of the molecular binding pocket. (L) Two-dimensional interaction map for XANA onto AhR protein binding site. (M) Gibbs energy landscape (2D). (N) Gibbs energy landscape (3D). (O) RMSD. (P) RMSF.
